# Microbiota of vaccinated and non-vaccinated clinically inconspicuous and conspicuous piglets under natural *Lawsonia intracellularis* infection

**DOI:** 10.3389/fvets.2022.1004506

**Published:** 2022-10-27

**Authors:** Julia Hankel, Saara Sander, Uthayakumar Muthukumarasamy, Till Strowig, Josef Kamphues, Klaus Jung, Christian Visscher

**Affiliations:** ^1^Institute for Animal Nutrition, University of Veterinary Medicine Hannover, Foundation, Hanover, Germany; ^2^Helmholtz Center for Infection Research, Brunswick, Germany; ^3^Hannover Medical School, Hanover, Germany; ^4^Genomics and Bioinformatics of Infectious Diseases, Institute for Animal Breeding and Genetics, University of Veterinary Medicine Hannover, Foundation, Hanover, Germany

**Keywords:** intestinal pathogen, porcine proliferative enteropathy, oral vaccination, 16S rRNA gene, microbiome, *Prevotella*, *Collinsella*

## Abstract

*Lawsonia* (*L*.) *intracellularis* is a widespread, economically important bacterium causing the porcine proliferative enteropathy (PPE). In this study, we evaluated intestinal microbiota of naturally exposed *L. intracellularis*-positive pigs under standardized conditions. To obtain three independent repetitions, 27 *L. intracellularis-*infected pigs (19.0 ± 1.50 kg body weight) from one farm were divided into three groups at an age of 7 to 8 weeks (nine pigs/group). Pigs were either vaccinated against *L. intracellularis* via oral drenching on their 21st day of life (attenuated live vaccine) or non-vaccinated and selected according to clinical findings (pigs without deviating fecal consistency or with moderate to soft fecal consistency). Comparison of the clinically inconspicuous piglets that differed regarding their vaccination status showed fewer significant differences in fecal microbiota composition. The vaccination led to an overall enrichment of bacterial species belonging to the order *Clostridiales*, while species of the genus *Collinsella* and *Prevotella* were decreased. Several bacterial species belonging to the order *Bacteroidales*, mainly of the family *Prevotellacecae*, often closely matching *Prevotella copri* differed significantly between non-vaccinated clinically inconspicuous and conspicuous piglets. Whether those bacterial species play a role in mitigating the severity of an *L. intracellularis* infection remains to be defined.

## Introduction

*Lawsonia* (*L*.) *intracellularis* is an economically important bacterium and of major concern to the pig industry worldwide (1–7). Also widespread in European countries, the bacterium was detected in fecal samples of 90.3% of all sampled pig herds (3). *L. intracellularis* is the cause of the porcine proliferative enteropathy (PPE) (8). The clinical presentation can be acute, chronic or subclinical (9). Clinical signs depend on the form of the disease, which may range from mild to severe diarrhea, decreased feed consumption, and poor growth in case of the chronic form, the porcine intestinal adenomatosis (PIA), to sudden death in case of the acute form, the proliferative hemorrhagic enteropathy (PHE) (9, 10). These two clinically distinct forms of the disease differ not only in severity and clinical symptoms, but also with regard to the occurrence at a particular age, while PIA is the most common form diagnosed usually between 6 and 20 weeks of age (9). Even though pigs with subclinical PPE have no detectable clinical signs, they show reduced weight gain during the growth and fattening period (9) and in all forms, a proliferation of intestinal epithelial cells containing intracellular *L. intracellularis* can be found (11).

While *L. intracellularis* is the cause of disease, it is not possible to rule out the interaction of one or more additional bacteria required for disease (12). Contact to the pathogen must not necessarily always lead to clinical disease. Gnotobiotic pigs lacking a normal intestinal microbiota were not colonized by the organism and failed to develop lesions (13). Further studies indicate that the intestinal microbiota of pigs are influenced by the infection with *L. intracellularis* itself (12, 14, 15). A clear change in community structure was observed at 21 and 28 days after experimental infection in both the small and large intestine of pigs (12). In addition, serum concentrations of folate and cobalamin, which can be of dietary origin or supplied from biosynthesis by distal gut microbiota (16, 17), were lower in pigs with PIA compared to pigs with the subclinical form (18).

Oral vaccination with an attenuated *L. intracellularis* strain (Enterisol^®^ Ileitis) is helpful for counteracting the disease, but does not prevent infection or transmission of the bacterium (19–23). Enterisol^®^ Ileitis is a licensed oral, live-attenuated vaccine that confers reduction of intestinal lesions caused by *L. intracellularis*, growth variability and loss of weight associated with the disease. Recent findings show that oral vaccination with Enterisol^®^ Ileitis seems to additionally alter the intestinal microbiota (12, 24, 25). Finally, an even and diverse microbiota community seems to benefit pigs infected with *L. intracellularis* (26). Based on these findings, an interaction of *L. intracellularis* and the present intestinal microbiota or their metabolites can be assumed.

The aim of the present evaluation was to compare fecal microbiota of vaccinated piglets, non-vaccinated piglets without clinical symptoms, and non-vaccinated piglets with moderate clinical symptoms to address two questions: First, whether vaccination alters intestinal microbiota under naturally occurring infection, and second, whether microbiota differ between non-vaccinated animals with and without clinical symptoms. Results from this evaluation may help to better understand potential interactions of the host microbiota and *L. intracellularis* and might identify bacteria that are potentially related to the protection against a clinical onset of the disease.

## Materials and methods

The experiment was approved by the Animal Welfare Officer of the University of Veterinary Medicine Hannover, Germany (reference: TiHo-T-2012-13).

### Experimental design, animals housing and sampling

The investigations took place in three independent repetitions (Rep 1–3), where in total, 27 piglets were reared under the same conditions. The piglets were obtained from one farm with 420 sows of Danish genetics (DK: Danish Landrace 50% × Yorkshire 50%). Sows were regularly vaccinated (Porcine Reproductive and Respiratory Syndrome Virus (PRRSV), Swine Influenza Virus, Porcine Parvovirus, *Erysipelothrix rhusiopathiae, Escherichia coli* + *Clostridium perfringens* dam vaccine) as were the piglets (DK x Pietrain) against Porcine Circovirus 2, *Mycoplasma hyopneumoniae*, and *Glaesserella parasuis*. This farm was system partner in a regional health-monitoring program including a regular monitoring scheme. Every 6 month a farm is sampled. Among others, blood samples are tested for PRRSV, *Salmonella* and porcine circovirus 2, and bulk samples of feces are formed and tested for *Salmonella, Brachyspira hyodysenteriae, Brachyspira pilosicoli*, and *Lawsonia*. In the context of such a regular and long-term screening program, the herd showed no signs of *Salmonella* and *Brachyspira* infections, but clinical symptoms of an *L. intracellularis* infection were a common finding in piglets. Confirmed by pathogen detection in feces, alterations in fecal consistency normally occurred at about an age of 7 to 9 weeks. In contrast to many other *L. intracellularis* infection studies in which pigs are often experimentally challenged with gut homogenates containing besides *L. intracellularis* other bacteria or viruses (12) that could affect microbiota composition, an experimental approach was chosen to best reflect natural infection examined under standardized conditions.

At 2 week intervals, approximately 50 piglets per trial from a total of four to five complete litters were vaccinated via oral drenching on their 21st day of life with a commercially available inactivated *L. intracellularis* vaccine strain (Enterisol^®^Ileitis, Boehringer Ingelheim Vetmedica GmbH, Ingelheim/Rhine, Germany). Subsequently, the piglets were marked individually.

**Figure 1:**
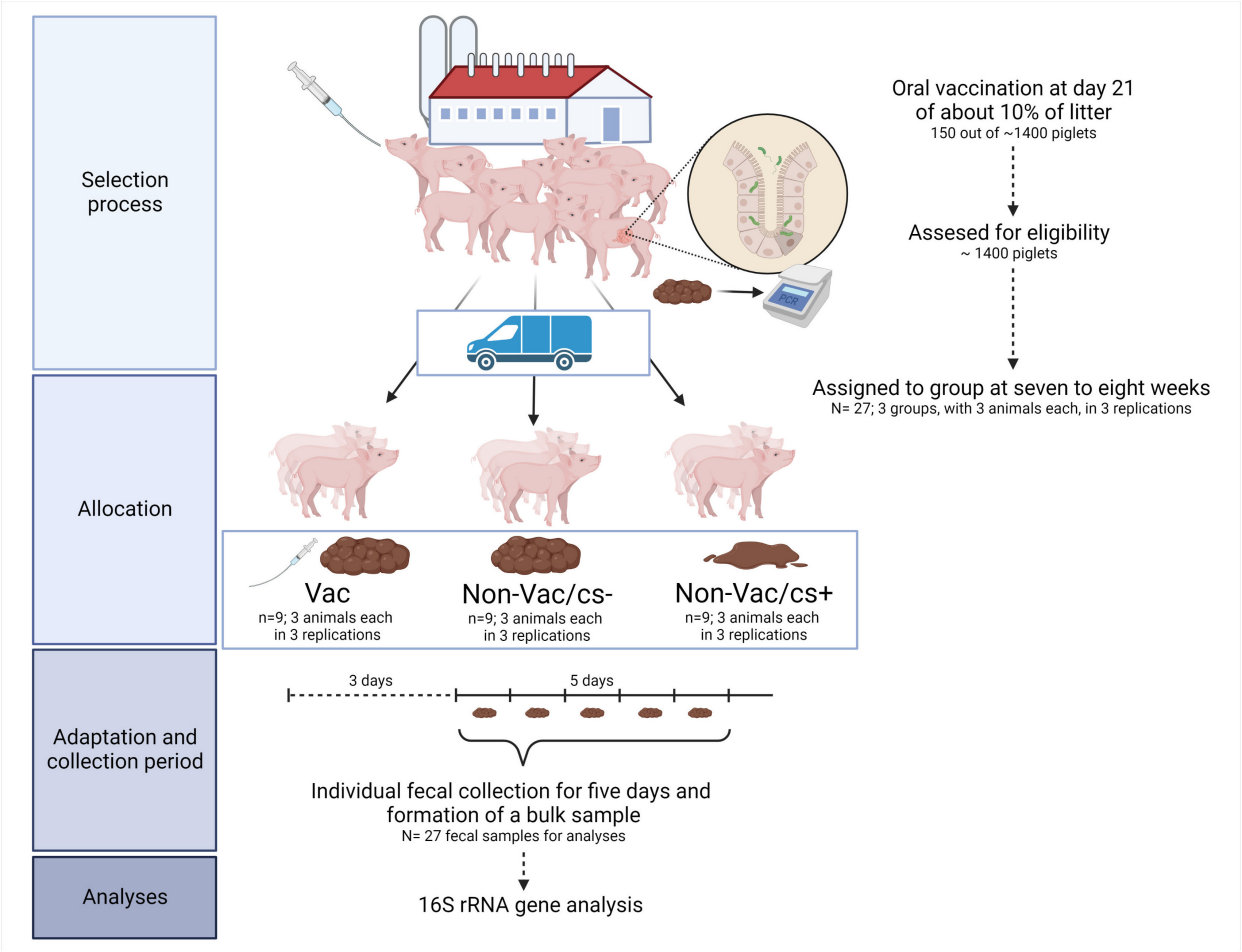
Clinical trial flow diagram. The diagram indicates selection process, the trial set-up, the collection of samples, and the analyzed parameters. The figure was created with Biorender.com.

At weaning, all 450 to 500 piglets were reared mixed (non-vaccinated and vaccinated animals) in groups of 12 to 30 animals. From weaning onwards, samples were taken on group basis regularly and tested for their *L. intracellularis* status by real-time PCR according to established methods (27). At the very moment when *L. intracellularis* was detected in one sample, a single-animal examination procedure took place and all piglets in a weaning group were clinically examined. Three conspicuous animals (pigs with moderate to soft fecal consistency, no siblings) were randomly taken from the appropriate weaning group (Non-vac/cs+). At the same time, three inconspicuous animals (without deviating fecal consistency, Non-vac/cs–) and three vaccinated animals (without deviating fecal consistency, Vac) of the same age and identical weight were randomly chosen in a balanced gender relationship (Figure 1). *L. intracellularis* was detected in fecal samples of all selected pigs by real-time PCR at this time. Thus, animals were selected for the study at an early stage of infection and not at a later stage when symptoms become more apparent and might also have an increasing impact on intestinal microbiota composition. All selected animals, 14 female and 13 castrated male pigs at an age of 7 to 8 weeks with a mean body weight of 19.0 ± 1.50 kg, were transported to the Institute for Animal Nutrition, University of Veterinary Medicine Hannover, Foundation, Hannover, Germany.

At the Institute for Animal Nutrition, the pigs were fed the diet they had been accustomed to on the farm. The complete diet for all trial replications derived from one batch and was offered *ad libitum*. The nutrient composition was in accordance with the official recommendation for piglets in Germany. The diet consisted of wheat, barley, soybean meal, soybean oil, and of a mineral and vitamin supplement, and contained 20.1% crude protein on dry matter basis and 13.8 MJ ME per kg diet.

Subsequent to a 3 day adaptation period, the total daily amount of feces was collected on 5 consecutive days. A bulk sample was formed from feces of the 5 day collection period, from which, subsequent to homogenization, an aliquot was taken for microbiota analysis, and determination of *L. intracellularis* genome equivalents (GE, shown in the logarithm of 10) via quantitative PCR according to established methods (27). Fecal shedding of *L. intracellularis* was found in all groups; in total, still 25 of all 27 piglets excreted *L. intracellularis*. The mean fecal excretion of *L. intracellularis* did not differ significantly, only numerically, between the chosen animals of the groups in the present study (22), thus making the groups comparable for comparisons to identify bacteria potentially contributing to the clinical outcome of the infection.

### DNA extraction, sequencing and data processing

16S rRNA gene analyses were performed as described in Hankel (28). Samples were first purified (Kit: BS 365, BioBasic Inc., Ontario, Canada) before hypervariable region V4 of the 16S rRNA gene was amplified according to previously described protocols (29) using primer F515/R806. Amplicons were sequenced with the Illumina MiSeq platform (PE250) and Usearch8.1 software package (http://www.drive5.com/usearch/) was used to assemble, quality control and cluster obtained reads. The command “fastq_mergepairs” with argument “fastq_maxdiffs 30” was used to merge the reads. Chimeric sequences were identified and removed with the help of the command “cluster_otus” (-otu_radius_pct 3) and the Uchime command included in the Usearch8.1 workflow. Quality filtering was set up with “fastq_filter” (-fastq_maxee 1) accepting a minimum read length of 200 bp. Reads were clustered into 97% ID operational taxonomic units (OTUs) and the UPARSE algorithm (30) was used to determine the OTU clusters and representative sequences. Taxonomy assignment was performed with the help of Silva database v128 (31) and the Naïve Bayesian Classifier from the Ribosomal Database Project (RDP) (32) with a bootstrap confidence cutoff of 70%.

OTUs that were not present in more than at least one sample were pruned and OTUs with an abundance < 0.02% were filtered. Finally, samples with fewer than 999 total reads were removed and reads assigned to chloroplast and mitochondria were filtered. After these filtering steps, all 27 samples could be included in the statistical analysis. The dataset contained 181,046 reads (mean number of reads: 6,705; range: 2,757–13,475) mapped to 124 OTUs.

### Statistical analysis

Data visualization and statistical analyses of microbiota were performed with R (version 4.1.2, www.r-project.org) using the R-packages “phyloseq” (version 1.36.0) (33). Selected alpha diversity indices (Observed, Chao 1, and Shannon) were also calculated with “phyloseq.” Means of alpha diversity estimates were compared with the aim to evaluate the influence of the factor Group. Data were checked for normality by analyzing the model residuals with the Shapiro-Wilk normality test implemented in the package “rstatix” [version 0.7.0 (34)], before conducting multiple and pairwise comparisons. Total community structure and composition of samples taken during the whole experimental phase were assessed for changes in relation to the experimental repetition and the treatments by permutational multivariate analysis of variance using Bray-Curtis distance (PERMANOVA) via the adonis function of the “vegan” package (version 2.5.7) (35). Ordination was performed using the Bray–Curtis dissimilarity-based principal coordinate analysis (PCoA). Differentially abundant OTUs between the groups were identified with the help of the R-package “DESeq2” (version 1.32.0), which uses tests based on the negative binomial distribution (36). Raw *p*-values were adjusted using the method of Benjamini and Hochberg (37) to control a false discovery rate (FDR) of 5%. Additionally, a cutoff for the log2-fold change of ±1 was set. Volcano plots were used to visualize differentially abundant OTUs. Statements of statistical significance were based upon *p*-values < 0.05. Fecal *L. intracellularis* excretion (log_10_ GE) and relative abundance of *Prevotella* were additionally evaluated for association using Spearman's rank correlation.

## Results

### Intestinal microbiota

#### Alpha and beta diversity

**Figure 2:**
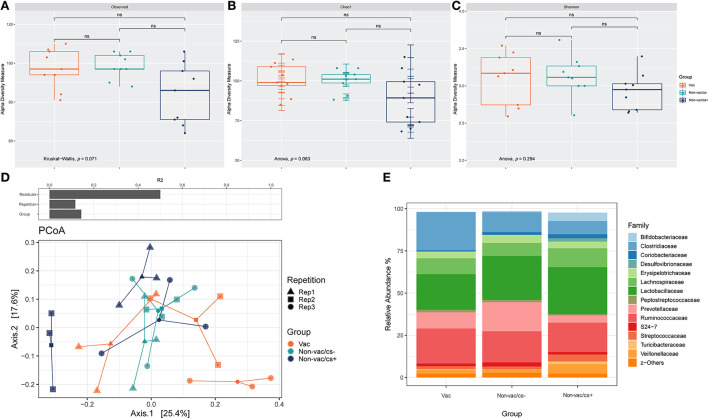
Box-plots showing alpha diversity in samples using the species richness estimators **(A)** Observed Species, **(B)** Chao1, and **(C)** Shannon index. **(D)** Permutational multivariate analysis of variance (PERMANOVA) on Bray–Curtis distances was used to quantify the contribution of the factors Repetition and Group to the differences in microbial composition of the samples (above). Ordination was performed using Bray-Curtis dissimilarity-based principal coordinate analysis (PCoA). Each point, square, and triangle represents a different piglet with inconspicuous (cs–) or conspicuous (cs+) *L. intracellularis* infection and different vaccination status (Vac and Non-vac) in three consecutive repetitions (Rep 1–3); colored lines connect samples of one treatment: Vac (orange), Non-vac/cs– (green) and Non-vac/cs+ (blue). **(E)** Bar charts represent the relative abundances of the 15 dominant families in fecal samples of piglets with different vaccination status (Vac and Non-vac) and inconspicuous (cs–) or conspicuous (cs+) *L. intracellularis* infection. Percentages do not total to 100% because only the 15 dominant families were considered.

Comparisons of measured species richness estimators, Observed Species, Chao 1 and Shannon index in feces of pigs revealed no statistically significant differences between the groups (Figures 2A–C).

Both the experimental repetition as well as the grouping had a significant effect on bacterial composition of the samples, while separation was pronounced due to grouping (Table 1, Figure 2D).

**Table 1:** Permutational multivariate analysis of variance (PERMANOVA) results based on Bray- Curtis dissimilarities.

	**DF**	**SumsOfSqs**	**F. Model**	***R* ^2^**	**Pr (>F)**
Repetition	2	0.3740	2.0954	0.11654	0.010 **
Group	2	0.4597	2.5753	0.14324	0.001 ***
Repetition: Group	4	0.7692	2.1544	0.23965	0.001 ***
Residuals	18	1.6066		0.50057	
Total	26	3.2095		1.00000	

**p-value < 0.01, ***p-value < 0.001.

#### Differences of bacterial abundance

The microbiota of feces were dominated at phylum level by *Firmicutes, Bacteroidetes*, and *Actinobacteria*. Relative abundances of families in fecal samples of animals within the three groups are shown in Figure 2E. Relative abundance of *Prevotellaceae* was highest in Non-vac/cs– (Non-vac/cs–: 17.2% ±7.0, Vac: 9.6% ±3.9, Non-vac/cs+: 4.2% ±2.3). Inversely, relative abundances of the family *Coriobacteriaceae* increased from vaccinated to non-vaccinated, clinically inconspicuous and non-vaccinated, clinically conspicuous animals (Vac: 0.7% ±0.5, Non-vac/cs–: 1.6% ±0.8, and Non-vac/cs+: 2.4% ±2.1).

**Figure 3:**
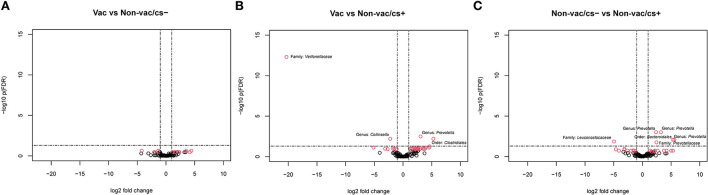
Volcano plot visualizing –log10 FDR-adjusted *p*-values vs. log2-fold changes for all 124 OTUs between **(A)** Vac and Non-vac/cs–, **(B)** Vac and Non-vac/cs+, and **(C)** Non-vac/cs− and Non-vac/cs+. The horizontal lines show significance threshold for the adjusted *p*-values < 0.05. Each open point represents a single OTU. Open points above the significance threshold and beyond the log2-fold change criterion of ±1 are indicated in red and bacterial taxonomy is labeled.

The number of differentially abundant OTUs in pairwise comparisons are shown in Table 2. Volcano plots visualize differentially abundant OTUs in Figure 3. Comparison of the clinically inconspicuous piglets that differed regarding their vaccination status (Vac vs. Non-vac/cs–) showed fewer significantly different OTUs. None met the selected criterion of an FDR-adjusted *p*-value < 0.05 and an absolute log2-fold change >1 (Figure 3A). Nevertheless, still 13 of 124 OTUs were significantly (*p*-values < 0.05 and absolute log2-fold change >1) different between Vac and Non-vac/cs–. Of a total of eight significantly enriched OTUs due to vaccination, sequences of seven OTUs belonged to the order *Clostridiales* (Supplementary Table 1). The only sequences of OTUs that decreased due to vaccination belonged to the genus *Prevotella*, closely matching *Prevotella copri*, and the family *Coriobacteriaceae* from which one OTU closely matched *Collinsella aerofaciens* (OTU_ID 4481613).

**Table 2:** Number of differentially abundant OTUs in the pairwise comparisons.

**Comparison**	**# *p* < 0.05**	**# pFDR < 0.05**	**# pFDR < 0.05 and abs (log2FC) > 1**
Vac vs. Non-vac/cs–	13	0	0
Vac vs. Non-vac/cs+	36	4	4
Non-vac/cs- vs. Non-vac/cs+	25	6	6

Raw *p*-values were adjusted using the method of Benjamini and Hochberg (37) to control a false discovery rate (FDR) of 5%. Additionally, a cutoff was set for the log2-fold change of ±1.

At species level, four of 124 OTUs showed significantly (selected with a criterion of FDR-adjusted *p*-values < 0.05 and absolute log2-fold change >1) different abundant sequences between Vac and Non-vac/cs+ (Figure 3B, Supplementary Table 2). Two OTUs were enriched in fecal samples of non-vaccinated and clinically conspicuous animals: one bacterial member of the family *Veillonellaceae* (OTU 300829) and the second, again OTU 4481613 (genus *Collinsella* within the family *Coriobacteriaceae*, closely matching *Collinsella aerofaciens*). In contrast, sequences of one OTU belonging to the genus *Prevotella*, family *Prevotellaceae*, were enriched in vaccinated compared to non-vaccinated and clinically conspicuous animals. This OTU closely matched the species *Prevotella copri* (OTU_ID 180825).

Comparison of both non-vaccinated groups of piglets that differed regarding their clinical outcome showed significantly different abundant sequences of six OTUs (selected with a criterion of FDR-adjusted *p*-values < 0.05 and absolute log2-fold change >1), of which five were enriched in clinically inconspicuous animals (Non-vac/cs–) compared to clinically conspicuous animals (Non-vac/cs+, Figure 3C, Supplementary Table 3). All five OTUs belonged to the order *Bacteroidales*, four of the five to the family *Prevotellaceae* and again three of the four OTUs closely matched the species *Prevotella copri*. The most enriched OTU that closely matched the species *Prevotella copri* was again OTU_ID 180825, already found to be enriched in vaccinated compared to non-vaccinated and clinically conspicuous animals (as mentioned above), while both other OTUs were decreased in Vac compared to Non-vac/cs–. Only one OTU (OTU_64384) belonging to the family *Leuconostocaceae* was enriched in samples of clinically conspicuous animals (Non-vac/cs+) compared to clinically inconspicuous animals (Non-vac/cs–).

Spearman's rank correlation revealed no relation between fecal *L. intracellularis* excretion (log_10_ GE) and the relative abundance of the genus *Prevotella* in clinically inconspicuous animals (Non-vac/cs– and Vac; correlation coefficient: −0.127; *p* > 0.05), while a significant relation was found in animals of the non-vaccinated groups (Non-vac/cs– and Non-Vac/cs+; correlation coefficient: −0.490; *p* < 0.05).

## Discussion

### Intestinal microbiota and severity of *L. intracellularis* infection

Fecal bacterial diversity was not significantly different between the groups of the present study. Still, all measured richness estimators of non-vaccinated, clinically conspicuous pigs were numerically lower compared to both the vaccinated and the non-vaccinated clinically inconspicuous pigs. Muwonge (26) observed an increased diversity of ileal microbiota associated with a reduction in *L. intracellularis* shedding. It has to be taken into account that the sampling sites differed in both studies and it is quite possible that there were pronounced differences in microbiota diversity within the ileum of the pigs that could no longer be clearly visualized in feces.

Significant differences in the bacterial composition of fecal samples between the three groups were observed in the present study (*p* = 0.001, *R*^2^ = 0.1432), while the distinction between group microbial communities was higher compared to the study of Leite et al. (12). At 3 weeks post-vaccination, a significant difference in community structure was found in feces of pigs due to oral live vaccine against *L. intracellularis* (Bray–Curtis dissimilarity PERMANOVA: *p* = 0.042, *R*^2^ = 0.041) (12).

The comparison of the clinically inconspicuous piglets (vaccinated or not) with clinically conspicuous piglets repeatedly revealed differences concerning OTUs belonging to the genus *Prevotella* within the family *Prevotellaceae* (phylum *Bacteroidetes*). Sequences of OTU_180825 (closely matched the species *Prevotella copri*) showed an 8-fold decrease (log2-fold change of about 3) in abundance of sequences in clinically conspicuous animals compared to both vaccinated or non-vaccinated clinically inconspicuous animals. In addition, spearman's rank correlation revealed a relation between fecal *L. intracellularis* excretion (log_10_ GE) and the relative abundance of the genus *Prevotella* in animals of the non-vaccinated groups (Non-vac/cs– and Non-Vac/cs+; correlation coefficient: −0.490; *p* < 0.05). Two further OTUs that also closely matched this species, *Prevotella copri*, were found to be significantly enriched in non-vaccinated clinically inconspicuous compared to conspicuous pigs. However, as described above, both OTUs were decreased due to vaccination in comparison to vaccinated and non-vaccinated clinically inconspicuous animals. It should be highlighted that 16S rRNA gene sequencing do not provide a reliable taxonomic resolution at species level and especially in case of *Prevotella copri*, even being the most predominant species in the gastrointestinal tract of adult pigs, a genus level classification is insufficient, which prompts the need for characterizing the role of most common *Prevotella* species in pigs using metagenomic and culture dependent approaches (38). Within *Bacteroidetes*, members of the genus *Prevotella* emerged as an underexplored keystone species within the human and animal microbiome (38, 39). *Prevotella* is highly interactive with other bacterial species in the gut (40, 41), and those highly connected taxa have been termed as keystone taxa that drive the microbiome structure and function irrespective of their abundance (42). *Prevotella* was identified as the most predominant genus within the intestinal tract of pigs, being an important member of both the upper and lower gastrointestinal tract microbiota (40, 43, 44) and a central constituent in one of the two most common bacterial enterotypes of pigs after weaning (41, 45–47). Mucosal microbiota of the ileum in adult pigs harbor significantly greater abundance of *Prevotella* compared to luminal microbiota in the same site (44). Significant differences in abundance and dynamics at sub-OTU level within this genus were observed in pigs pre- and post-weaning, while *Prevotella copri*, likely to be vertically transmitted but suppressed during the suckling stage, dramatically increase in the gastrointestinal tract at the end of the nursery phase after the introduction of solid food and gradually decrease at subsequent stages (40). *Prevotella* (particularly *Prevotella copri*) might be a critical bacterial taxon stimulating the feed intake in pigs (45) and was significantly associated with fat accumulation (48) and growth performance of pigs after weaning (41, 46). There is currently no proposed or demonstrated mechanism through which *Prevotella* increases feed intake, and *Prevotella* enrichment may be a product of increased feed intake rather than its driver (38). Also in the present study, the average daily feed intake (Vac: 1.297 ± 0.116, Non-vac/cs–: 1.207 ± 0.119, Non-vac/cs+: 1.165 ± 0.148 kg dry matter/day) and average daily weight gain (Vac: 894^a^ ± 73.3, Non-vac/cs−: 857^ab^ ± 86.3, Non-vac/cs+: 785^b^ ± 137 g/day) differed numerically between both non-vaccinated groups (22). *In vitro* studies have repeatedly demonstrated that gut microbiomes rich in *Prevotella* show higher complex polysaccharides utilizing capacity when compared to a *Bacteroides*-dominated population with significantly higher concentrations of SCFAs, especially propionate, which may in part explain why *Prevotella*-driven enterotype is believed to benefit host health (49). Feeding diets with high amylose-to-amylopectin ratio to finishing pigs with *Prevotella*-rich enterotype, rather than driven by *Bacteroides*, increases the number and activity of butyrate-producing bacteria and the concentration of total SCFA, which may benefit gut health due to potential decreased expression of mucosal inflammation associated genes (49). Members of the genus *Prevotella* were associated with positive outcomes in pig production, not only with regard to growth performance but also to immune response (38). Compared to the *Ruminococcaceae* enterotype, the *Prevotella*-dominant enterotype has been associated with higher production of secretory IgA in adult pigs (46), which serves as the first line of innate defense against invading pathogens (50) and also probably plays an important role in protecting the intestine against *L. intracellularis* invasion and intracellular proliferation (51). However, it is also possible that *Prevotella* does not induce an IgA response, but simply benefits from elevated levels of secretory IgA (38). In addition, *Prevotella copri* is a vitamin B producer and possesses a folate and cobalamin biosynthesis pathway (17). Serum concentrations of folate and cobalamin were found to be lower in pigs with PIA compared to those having the subclinical form (18). The authors discussed that this can have an effect on amino acid metabolism and nucleic acid synthesis (18). On the other hand, gut microbiota might modulate host immune function via B vitamins (17). Dietary vitamin B9 (folate) deficiency resulted in a reduction in the regulatory T cell population which plays an important role in the prevention of excessive immune responses, and mice fed a vitamin B9-deficient diet exhibit increased susceptibility to intestinal inflammation [reviewed in (17)]. Dietary vitamin B12 (cobalamin) deficiency decreases the number of CD8^+^ T cells and suppresses natural killer T-cell activity in mice, suggesting that vitamin B12 contributes to the immune response via CD8^+^ T cells and natural killer T cells [reviewed in (17)]. CD8^+^ and CD4^+^CD8^+^ lymphocytes in pigs produce IFNγ, which is an important cytokine for protection against intracellular pathogens and suspected of being a crucial factor mediating protection against *L. intracellularis* infection (52). It has been shown that piglets that harbor higher *Prevotella* may have better protection against diarrhea. Judging by the results of a study conducted by Dou et al. (53), the higher abundance of *Prevotella* may contribute to allowing healthy pigs to better adapt to post-weaning dietary conditions, thereby reducing the risk of developing post-weaning diarrhea. Similar findings were seen by Sun et al. (54) who reported greater abundance of gut *Prevotella* in non-diarrheic piglets compared to diarrheic piglets, while *Prevotellaceae UCG-003* was the key bacterium in non-diarrheic microbiota of piglets. By using an ETEC (Enterotoxigenic *Escherichia coli)*-induced diarrheal model in piglets, it was shown that resistant piglets (challenged with ETEC but not suffering from diarrhea) demonstrated the highest percentage of *Prevotella* (6.7%) even compared to non-challenged control piglets (4.2%). In addition, *Prevotella* decreased as the piglets were transient from the pre-diarrheic state (1.7%) to the diarrheic state (0.2%) (55).

Whether bacterial species of the order *Bacteroidales* (especially *Prevotella*) play a role in mitigating the severity of the disease or are more an indicator of unimpaired feed intake and “balanced” ileal starch and protein digestibility rates, which maintain continuous fermentation conditions in the large intestine, remains to be defined. Muwonge et al. (26) found the top three families *Prevotellaceae, Ruminococcaceae*, and *Lactobacillaceae* as the core microbiota in their study to be associated with changes in the threshold for shedding *L. intracellularis*, but not to have a major effect on the clinical outcome of the disease. Some *Prevotella* species were found to be decreased due to oral *L. intracellularis* vaccination in the present study as well as in investigations by Leite et al. (12, 24), who found one *Prevotella* species to be increased in the former study and two *Prevotella* species to be increased and two to be decreased in the latter study due to oral *L. intracellularis* vaccination. *Prevotella* is a genus with high genetic diversity within and between species, and certain strains may exhibit pathobiontic properties by promoting chronic inflammation in humans (56, 57). Similar to pigs, the controversial role played by *Prevotella copri* in human health is discussed as well (56). As the *Prevotella* genus is certainly not exclusively beneficial, further research into the characterization at species or even strain level is needed to clarify its preventative or promotive role in the pathogenesis of pig diarrhea (38).

### Impact of oral vaccination on microbiota

To date, studies that evaluate whether oral immunization results in discernible alterations of the microbiota are rare (58, 59). Comparison of fecal microbiota between the vaccinated and non-vaccinated clinically inconspicuous piglets showed fewer differences compared to the other group comparisons in the present study. Nevertheless, the existing differences in terms of OTUs differing between the two groups (*Collinsella aerofaciens, Prevotella copri*, and *Clostridiales*) resemble previous investigations with experimental *L. intracellularis* challenge, that have also shown that oral vaccination against *L*. *intracellularis* shapes the gut microbiota in weaned piglets. Leite et al. (12, 24) who investigated the impact of an oral live vaccine against *L. intracellularis* (Enterisol^®^ Ileitis) in pigs experimentally challenged with *L. intracellularis* were in line with results of the present study. Vaccination against *L. intracellularis* in dually challenged pigs (*L. intracellularis* and *S. enterica* serovar Typhimurium) also induced a significant decrease in abundance of an OTU that closely matched *Collinsella aerofaciens* and a significant increase in the abundance of *Clostridium* species (24). In a further investigation by Leite et al. (12), vaccination led again to a significant decrease in the abundance of *Collinsella, Fusobacterium*, and *Campylobacter* among other microbial changes compared with non-vaccinated and *L. intracellularis*-challenged animals. The authors suggest that the intestinal pathogen challenge induces dysbiosis resulting from an increase in the number of such pathobionts and vaccination might mitigate these effects, which could contribute to the development and severity of disease (12). However, it must be emphasized that both studies characterized the porcine intestinal microbiota response of pigs to an experimental *L. intracellularis* challenge. Challenge material originates from *L. intracellularis* grown in McCoy cells (24), or mucosal scrapings from the ileum of pigs with confirmed *L. intracellularis* infection and gross PPE lesions in which *L. intracellularis* was the predominant bacterium (12). In the latter, other bacteria (species of *Campylobacter, Chlamydia trachomatis, Bacteroides fragilis*, and *Fusobacterium nucleatum*) detected in challenge material, along with some viruses, were enriched in the intestine of pigs challenged with a gut homogenate and affected by oral vaccination (12). The question remains whether these bacteria also play a role under natural infectious conditions, when evaluating potential effects of vaccination on microbiota. Therefore, studies investigating microbiome changes under naturally occurring *L. intracellularis* infection are still needed (12). To the best of our knowledge, the present study investigated microbiota of naturally exposed *L. intracellularis*-positive pigs under standardized conditions for the first time and could show similar microbiota changes due to vaccination for *Collinsella* and *Clostridiales* species as found in experimental *L. intracellularis* challenge models. Compared to vaccinated piglets, the OTU 4481613 (family *Coriobacteraceae*, genus *Collinsella*, closely matching *Collinsella aerofaciens*) was especially enriched in non-vaccinated clinically conspicuous animals, but also in non-vaccinated clinically inconspicuous animals. Leite et al. (12, 24), who observed similar results refer to this bacterium as pathobiont. Pathobionts are commensal bacteria that are typically kept at low levels within a healthy gut and do not cause problems in immune-competent hosts, but have the potential to cause harm to the host leading to inflammation and pathology (60). In human intestinal epithelial cell lines, it could be shown that *Collinsella* increases gut permeability by reducing the expression of tight junction protein and induces expression of IL-17 network cytokines, suggesting that an expansion of *Collinsella* may cause an increase in pro-inflammatory conditions with a loss of gut epithelial integrity (61). Augmented gut permeability is believed to result in an escape of bacteria and endotoxins from the intestinal lumen to the mesenteric lymph nodes and in portal circulation (62).

Comparison of vaccinated and non-vaccinated clinically conspicuous animals revealed besides *Collinsella* significant differences in sequences belonging to the family *Veillonellaceae*. Sequences of *Veillonellaceae* were enriched only in four of the nine animals belonging to the Non-vac/cs+ group (data not shown). The animal with the highest OTU sequence count belonging to *Veillonellaceae* had the highest fecal dry matter content in this group and at the same time within the range of clinically inconspicuous animals belonging to the other two groups (data not shown). For this reason, the enrichment in *Veillonellaceae* in Non-vac/cs+ does not appear to be related to disease severity. *Prevotellaceae, Veillonellaceae* as well as *Ruminococcaceae* and *Lachnospiraceae* very likely form a functional group in the lumen of healthy piglets, and the increased number of *Prevotella* within *Bacteroidetes* as well as of *Veillonellaceae, Lachnospiraceae* and *Ruminococcaceae* within the *Firmicutes* equip the large intestine with metabolic abilities that are indispensable for host survival; these families are positively correlated with gene functions related to amino acids, energy, cofactors and vitamins metabolism (63).

## Conclusion

Intestinal microbiota of pigs changed similarly due to oral *L. intracellularis* vaccination in naturally occurring *L. intracellularis* infection compared with experimental *L. intracellularis* infection.

In addition, intestinal microbiota differed between non-vaccinated animals with and without clinical symptoms presented as altered fecal quality. Sequences of several bacterial species belonging to the order *Bacteroidales*, mainly of the family *Prevotellacecae*, often closely matching *Prevotella copri*, were lower in non-vaccinated clinically conspicuous piglets compared to inconspicuous ones. Whether those bacterial species play a role in mitigating the severity of an *L. intracellularis* infection has yet to be determined.

## Data availability statement

The datasets presented in this study can be found in online repositories. The names of the repository/repositories and accession number(s) can be found at: https://www.ncbi.nlm.nih.gov/bioproject/?term=PRJNA862063.

## Ethics statement

The animal study was reviewed and approved by the Animal Welfare Officer of the University of Veterinary Medicine Hannover, Germany (reference: TiHo-T-2012-13).

## Author contributions

JH, JK, and CV: conceptualization. JH, SS, TS, JK, KJ, and CV: methodology. JH, SS, JK, KJ, and CV: validation. JH and KJ: formal analysis. SS and CV: investigation and supervision. JK: resources. JH, UM, KJ, and CV: data curation. JH: writing-original draft preparation and visualization. JH, JK, TS, KJ, and CV: writing-review and editing. SS, JK, and CV: project administration. JK and CV: funding acquisition. All authors have read and agreed to the published version of the manuscript. All authors contributed to the article and approved the submitted version.

## Funding

This Open Access publication was funded by the Deutsche Forschungsgemeinschaft (DFG, German Research Foundation) - 491094227 Open Access Publication Costs and the University of Veterinary Medicine Hannover, Foundation.

## Conflict of interest

The authors declare that the research was conducted in the absence of any commercial or financial relationships that could be construed as a potential conflict of interest.

## Publisher's note

All claims expressed in this article are solely those of the authors and do not necessarily represent those of their affiliated organizations, or those of the publisher, the editors and the reviewers. Any product that may be evaluated in this article, or claim that may be made by its manufacturer, is not guaranteed or endorsed by the publisher.
