# Supplementary material for: Microbiota of vaccinated and non-vaccinated clinically inconspicuous and conspicuous piglets under natural Lawsonia intracellularis infection
Source: Front Vet Sci. 2022 Oct 27;9:1004506. doi: 10.3389/fvets.2022.1004506 (PMC9648062; doi:10.3389/fvets.2022.1004506)
Supplement: Supplementary file 1 [file Data_Sheet_1.docx]

Supplementary Material

**Supplementary Table 1.** Differentially abundant OTUs in the pairwise comparisons of the groups Vac and Non-vac/cs-.

| **OTU_ID** | **log2FoldChange** | **pvalue** | **padj** | **Phylum** | **Class** | **Order** | **Family** | **Genus** | **Species** |
| --- | --- | --- | --- | --- | --- | --- | --- | --- | --- |
| 292921 | -4.23 | 0.0021 | 0.2322 | Bacteroidetes | Bacteroidia | Bacteroidales | Prevotellaceae | Prevotella | copri |
| 290284 | 4.43 | 0.0053 | 0.2322 | Firmicutes | Clostridia | Clostridiales | Ruminococcaceae | z-Others | z-Others |
| 4481613 | -1.35 | 0.0074 | 0.2322 | Actinobacteria | Coriobacteriia | Coriobacteriales | Coriobacteriaceae | Collinsella | aerofaciens |
| 25461 | 3.51 | 0.0089 | 0.2322 | Firmicutes | Clostridia | Clostridiales | Ruminococcaceae | Faecalibacterium | prausnitzii |
| 4436552 | -1.31 | 0.0094 | 0.2322 | Bacteroidetes | Bacteroidia | Bacteroidales | Prevotellaceae | Prevotella | copri |
| 4328026 | -2.07 | 0.0295 | 0.3474 | Actinobacteria | Coriobacteriia | Coriobacteriales | Coriobacteriaceae | z-Others | z-Others |
| 191792 | 1.21 | 0.0333 | 0.3474 | Firmicutes | Clostridia | Clostridiales | Ruminococcaceae | Faecalibacterium | prausnitzii |
| 351975 | 4.14 | 0.0338 | 0.3474 | Firmicutes | Clostridia | Clostridiales | Lachnospiraceae | z-Others | z-Others |
| 4372528 | 1.61 | 0.0380 | 0.3474 | Firmicutes | Clostridia | Clostridiales | Clostridiaceae | z-Others | z-Others |
| 782953 | 2.33 | 0.0392 | 0.3474 | Proteobacteria | Gammaproteobacteria | Enterobacteriales | Enterobacteriaceae | z-Others | z-Others |
| 33984 | 2.21 | 0.0413 | 0.3474 | Firmicutes | Clostridia | Clostridiales | z-Others | z-Others | z-Others |
| 4410166 | -1.26 | 0.0445 | 0.3474 | Bacteroidetes | Bacteroidia | Bacteroidales | Prevotellaceae | Prevotella | copri |
| 2318497 | 1.50 | 0.0457 | 0.3474 | Firmicutes | Clostridia | Clostridiales | Clostridiaceae | z-Others | z-Others |

Raw *p*-values were adjusted using the method of Benjamini and Hochberg to control a false discovery rate (FDR) of 5%.

**Supplementary Table 2.** Differentially abundant OTUs in the pairwise comparisons of the groups Vac and Non-vac/cs+.

| **OTU_ID** | **log2FoldChange** | **pvalue** | **padj** | **Phylum** | **Class** | **Order** | **Family** | **Genus** | **Species** |
| --- | --- | --- | --- | --- | --- | --- | --- | --- | --- |
| 300829 | -20.3 | 4.1E-15 | 5.0E-13 | Firmicutes | Clostridia | Clostridiales | Veillonellaceae | z-Others | z-Others |
| 180825 | 3.05 | 0.0001 | 0.0032 | Bacteroidetes | Bacteroidia | Bacteroidales | Prevotellaceae | Prevotella | copri |
| 33984 | 5.29 | 0.0002 | 0.0062 | Firmicutes | Clostridia | Clostridiales | z-Others | z-Others | z-Others |
| 4481613 | -2.23 | 0.0002 | 0.0062 | Actinobacteria | Coriobacteriia | Coriobacteriales | Coriobacteriaceae | Collinsella | aerofaciens |
| 4404187 | 4.64 | 0.0022 | 0.0543 | Firmicutes | Clostridia | Clostridiales | Lachnospiraceae | z-Others | z-Others |
| 178845 | 3.92 | 0.0040 | 0.0757 | Firmicutes | Clostridia | Clostridiales | Ruminococcaceae | z-Others | z-Others |
| 300859 | 1.94 | 0.0063 | 0.0757 | Bacteroidetes | Bacteroidia | Bacteroidales | Prevotellaceae | z-Others | z-Others |
| 235424 | 4.53 | 0.0068 | 0.0757 | Firmicutes | Clostridia | Clostridiales | Clostridiaceae | z-Others | z-Others |
| 825808 | -5.16 | 0.0070 | 0.0757 | Actinobacteria | Actinobacteria | Bifidobacteriales | Bifidobacteriaceae | z-Others | z-Others |
| 4372528 | 2.65 | 0.0072 | 0.0757 | Firmicutes | Clostridia | Clostridiales | Clostridiaceae | z-Others | z-Others |
| 185575 | 1.45 | 0.0075 | 0.0757 | Firmicutes | Clostridia | Clostridiales | Ruminococcaceae | Faecalibacterium | prausnitzii |
| 4468466 | 2.30 | 0.0083 | 0.0757 | Firmicutes | Clostridia | Clostridiales | Ruminococcaceae | z-Others | z-Others |
| 301251 | 2.94 | 0.0086 | 0.0757 | Bacteroidetes | Bacteroidia | Bacteroidales | Prevotellaceae | z-Others | z-Others |
| 663226 | -1.73 | 0.0087 | 0.0757 | Firmicutes | Clostridia | Clostridiales | Veillonellaceae | z-Others | z-Others |
| 70217 | 2.44 | 0.0121 | 0.0981 | Bacteroidetes | Bacteroidia | Bacteroidales | Prevotellaceae | z-Others | z-Others |
| 804995 | 3.48 | 0.0132 | 0.1003 | Bacteroidetes | Bacteroidia | Bacteroidales | S24-7 | z-Others | z-Others |
| 1013234 | 3.04 | 0.0146 | 0.1008 | Bacteroidetes | Bacteroidia | Bacteroidales | Prevotellaceae | z-Others | z-Others |
| 4374302 | -1.47 | 0.0153 | 0.1008 | Firmicutes | Clostridia | Clostridiales | Lachnospiraceae | Dorea | formicigenerans |
| 279235 | 1.69 | 0.0157 | 0.1008 | Firmicutes | Clostridia | Clostridiales | Peptostreptococcaceae | z-Others | z-Others |
| 3931537 | 2.69 | 0.0194 | 0.1031 | Firmicutes | Clostridia | Clostridiales | Clostridiaceae | z-Others | z-Others |
| 162660 | 3.46 | 0.0199 | 0.1031 | Firmicutes | Clostridia | Clostridiales | Ruminococcaceae | Ruminococcus | bromii |
| 185583 | 1.42 | 0.0212 | 0.1031 | Firmicutes | Clostridia | Clostridiales | Ruminococcaceae | Faecalibacterium | prausnitzii |
| 2318497 | 2.15 | 0.0214 | 0.1031 | Firmicutes | Clostridia | Clostridiales | Clostridiaceae | z-Others | z-Others |
| 4436552 | 1.16 | 0.0222 | 0.1031 | Bacteroidetes | Bacteroidia | Bacteroidales | Prevotellaceae | Prevotella | copri |
| 64384 | -3.12 | 0.0226 | 0.1031 | Firmicutes | Bacilli | Lactobacillales | Leuconostocaceae | z-Others | z-Others |
| 350503 | 2.29 | 0.0230 | 0.1031 | Firmicutes | Clostridia | Clostridiales | Ruminococcaceae | z-Others | z-Others |
| 693641 | 3.84 | 0.0234 | 0.1031 | Planctomycetes | Planctomycetia | Pirellulales | Pirellulaceae | z-Others | z-Others |
| 1959881 | 2.09 | 0.0237 | 0.1031 | Firmicutes | Clostridia | Clostridiales | Clostridiaceae | z-Others | z-Others |
| 290284 | 3.65 | 0.0265 | 0.1117 | Firmicutes | Clostridia | Clostridiales | Ruminococcaceae | z-Others | z-Others |
| 328717 | 2.06 | 0.0299 | 0.1144 | Firmicutes | Clostridia | Clostridiales | Lachnospiraceae | z-Others | z-Others |
| 1986324 | 2.07 | 0.0309 | 0.1144 | Firmicutes | Clostridia | Clostridiales | Clostridiaceae | z-Others | z-Others |
| 3025 | -2.69 | 0.0311 | 0.1144 | Chlamydiae | Chlamydiia | Chlamydiales | Chlamydiaceae | z-Others | z-Others |
| 4469164 | -1.70 | 0.0312 | 0.1144 | Tenericutes | Mollicutes | RF39 | z-Others | z-Others | z-Others |
| 2157225 | 2.23 | 0.0327 | 0.1144 | Firmicutes | Clostridia | Clostridiales | Clostridiaceae | z-Others | z-Others |
| 4295707 | -1.10 | 0.0328 | 0.1144 | Firmicutes | Erysipelotrichi | Erysipelotrichales | Erysipelotrichaceae | Eubacterium | biforme |
| 4326870 | 2.61 | 0.0353 | 0.1196 | Firmicutes | Clostridia | Clostridiales | Veillonellaceae | z-Others | z-Others |

Raw *p*-values were adjusted using the method of Benjamini and Hochberg to control a false discovery rate (FDR) of 5%.

**Supplementary Table 3.** Differentially abundant OTUs in the pairwise comparisons of the groups Non-vac/cs- and Non-vac/cs+.

| **OTU_ID** | **log2FoldChange** | **pvalue** | **padj** | **Phylum** | **Class** | **Order** | **Family** | **Genus** | **Species** |
| --- | --- | --- | --- | --- | --- | --- | --- | --- | --- |
| 180825 | 3.22 | 1.5E-05 | 0.0010 | Bacteroidetes | Bacteroidia | Bacteroidales | Prevotellaceae | Prevotella | copri |
| 4436552 | 2.38 | 1.6E-05 | 0.0010 | Bacteroidetes | Bacteroidia | Bacteroidales | Prevotellaceae | Prevotella | copri |
| 292921 | 5.57 | 0.0002 | 0.0083 | Bacteroidetes | Bacteroidia | Bacteroidales | Prevotellaceae | Prevotella | copri |
| 804995 | 5.33 | 0.0003 | 0.0083 | Bacteroidetes | Bacteroidia | Bacteroidales | S24-7 | z-Others | z-Others |
| 64384 | -4.94 | 0.0006 | 0.0136 | Firmicutes | Bacilli | Lactobacillales | Leuconostocaceae | z-Others | z-Others |
| 300859 | 2.43 | 0.0009 | 0.0175 | Bacteroidetes | Bacteroidia | Bacteroidales | Prevotellaceae | z-Others | z-Others |
| 328458 | -3.13 | 0.0062 | 0.1095 | Firmicutes | Bacilli | Lactobacillales | Streptococcaceae | Streptococcus | z-Others |
| 248902 | -4.61 | 0.0083 | 0.1270 | Firmicutes | Bacilli | Turicibacterales | Turicibacteraceae | z-Others | z-Others |
| 193672 | -3.14 | 0.0101 | 0.1384 | Firmicutes | Clostridia | Clostridiales | Clostridiaceae | z-Others | z-Others |
| 46910 | -1.58 | 0.0138 | 0.1694 | Firmicutes | Clostridia | Clostridiales | Ruminococcaceae | z-Others | z-Others |
| 4404187 | 5.32 | 0.0166 | 0.1844 | Firmicutes | Clostridia | Clostridiales | Lachnospiraceae | z-Others | z-Others |
| 22466 | 4.22 | 0.0183 | 0.1844 | Bacteroidetes | Bacteroidia | Bacteroidales | Prevotellaceae | z-Others | z-Others |
| 4326870 | 2.18 | 0.0207 | 0.1844 | Firmicutes | Clostridia | Clostridiales | Veillonellaceae | z-Others | z-Others |
| 3931537 | 2.66 | 0.0219 | 0.1844 | Firmicutes | Clostridia | Clostridiales | Clostridiaceae | z-Others | z-Others |
| 918242 | -1.99 | 0.0240 | 0.1844 | Cyanobacteria | 4C0d-2 | YS2 | z-Others | z-Others | z-Others |
| 279235 | 1.36 | 0.0250 | 0.1844 | Firmicutes | Clostridia | Clostridiales | Peptostreptococcaceae | z-Others | z-Others |
| 3039313 | -2.00 | 0.0268 | 0.1844 | Firmicutes | Clostridia | Clostridiales | Veillonellaceae | z-Others | z-Others |
| 368490 | -2.59 | 0.0271 | 0.1844 | Firmicutes | Bacilli | Turicibacterales | Turicibacteraceae | z-Others | z-Others |
| 178845 | 3.77 | 0.0312 | 0.1844 | Firmicutes | Clostridia | Clostridiales | Ruminococcaceae | z-Others | z-Others |
| 300829 | -4.09 | 0.0313 | 0.1844 | Firmicutes | Clostridia | Clostridiales | Veillonellaceae | z-Others | z-Others |
| 287790 | 5.03 | 0.0320 | 0.1844 | Firmicutes | Clostridia | Clostridiales | Ruminococcaceae | z-Others | z-Others |
| 237444 | -1.46 | 0.0330 | 0.1844 | Firmicutes | Bacilli | Lactobacillales | Streptococcaceae | Streptococcus | luteciae |
| 4481613 | -1.20 | 0.0384 | 0.2053 | Actinobacteria | Coriobacteriia | Coriobacteriales | Coriobacteriaceae | Collinsella | aerofaciens |
| 572743 | 2.07 | 0.0428 | 0.2195 | Bacteroidetes | Bacteroidia | Bacteroidales | Prevotellaceae | z-Others | z-Others |
| 110660 | 1.60 | 0.0499 | 0.2397 | Firmicutes | Clostridia | Clostridiales | z-Others | z-Others | z-Others |

Raw *p*-values were adjusted using the method of Benjamini and Hochberg to control a false discovery rate (FDR) of 5%
